# A Study on the Pre-Hardened Shrinkage Reduction of Grout Using Carbon Materials

**DOI:** 10.3390/ma17153775

**Published:** 2024-08-01

**Authors:** Jeong-Bae Lee, Seong-Soo Kim, Young-Jun Lee, In-Soo Jang, Ju-Youn Kim

**Affiliations:** 1 Department of Smart Construction and Environmental Engineering, Daejin University, Pocheon 11159, Republic of Korea; jblee@daejin.ac.kr (J.-B.L.); sskim@daejin.ac.kr (S.-S.K.); 2 Department of Civil and Environmental Engineering, Daejin University, Pocheon 11159, Republic of Korea; iwjn123@naver.com (Y.-J.L.); wkddlstn70@naver.com (I.-S.J.)

**Keywords:** cement expansive agent, carbon materials, charcoal, grout expansion, pre-hardening shrinkage

## Abstract

In this study, the characteristics of grout mixed with charcoal as an expansive agent were examined to reduce the pre-hardening shrinkage of cementitious materials. This study compared and reviewed the application of CSA, a conventional expansive agent, to grout. The setting time, fluidity, compressive strength, and pre-hardening shrinkage/expansion were evaluated to explore the usability of charcoal as an expansive agent. The test results confirmed that, as the incorporation rate of charcoal increased, the pre-hardening expansion rate of the grout also increased, making it more effective for pre-hardening expansion than the conventional expansive agent CSA. However, when charcoal was used as an expansive agent, the compressive strength decreased after hardening, indicating the need for caution regarding the amount of charcoal used. Furthermore, the pre-hardening shrinkage and expansion rates of the various types of charcoal used in this study showed some differences, suggesting the need for further research on the internal pore volume and pore size of the charcoal.

## 1. Introduction

Recently, there have been instances of tendon failure in domestic PSC (prestressed concrete) bridges, and the cause has been identified as corrosion due to moisture infiltration resulting from grout shrinkage [1,2].

Cementitious materials are a type of construction material that produce hydration reactions inevitably leading to shrinkage [3,4,5,6], such as plastic shrinkage due to the loss of moisture from an early stage [3,7,8], autogenous shrinkage due to chemical reactions during the hydration process [9,10], and drying shrinkage due to the evaporation of excess water into the external environment after hardening [3,11,12,13,14,15,16,17]. Such shrinkage creates tensile stress on the surface of the hardened paste. If the tensile stress exceeds the tensile strength of the hardened paste, cracks occur which reduce the durability of the cementitious material [18], increase the repair and reinforcement costs, and decrease the service life of the building structures [19,20].

Grout is used as a filler material in bridge bearings, the foundations of machines, and prestressed concrete bridge ducts [1,2]. Hence, the type of grout used must ensure the integrity of building structures by reducing shrinkage to disperse stress and protect the reinforcing bars from deterioration factors [1,2].

Expansive agents are used to reduce the shrinkage of grout [21], and calcium sulfoaluminate (CSA) and aluminum powder are the most commonly used expansive agents [13,14,22,23,24]. The expansion property of CSA is attributed to the creation of needle-like ettringite (C_3_A.3CaSO_4_.32H_2_O) over a period of several hours via a hydration reaction with water [14,23,25,26,27]. Hence, CSA is effective against the drying shrinkage that occurs after hardening [28,29,30]. However, it cannot effectively reduce the pre-hardening shrinkage in the plastic state [31,32]. Moreover, aluminum powder exhibits its expansion property within the cementitious materials in their plastic states by generating pores caused by hydrogen gas, while the cementitious materials are mixed. However, as extremely small amounts of aluminum power are used, cracks and structural damage can occur due to partial or excessive expansion, thus making quality control difficult. These chemically reactive expansion agents are sensitive to various factors [33], such as the temperature of the cementitious material, the hardening speed, and the curing method, which can affect the expansion performance [27,34,35,36,37]. Therefore, more effective expansive agents are needed [38,39,40,41].

To overcome the drawbacks of chemical-reaction–based expansive agents [42], this study focused on examining materials that exhibit physical expansion and verifying their expansion properties [43,44]. This study utilized porous carbon materials, which release air and physically expand when they come into contact with moisture. Therefore, these materials are less sensitive to the temperature, curing method, and hardening speed than chemical-reaction–based expansion agents and demonstrate consistent expansive performance [45].

Carbon materials with physical expansion properties have numerous pores on their surfaces and inside the materials. Hence, these materials can absorb water from the hardened cementitious paste and release an equivalent amount of air inside the material. This characteristic allows the material to exhibit its expansion property within several minutes in a hardened cementitious paste [46]. Furthermore, the expansion property can be secured when the material is in a plastic state before hardening.

Therefore, this study evaluated the pre-hardening shrinkage and expansion characteristics of grout mixed with a carbon material with a physical expansion mechanism to secure non-shrinkage properties by reducing the pre-hardening shrinkage of the grout. In addition, the setting time, flow time, and compressive strength were compared to examine the physical properties of grout mixed with carbon-based expansive agents. For comparison, the physical properties of grout mixed with CSA were evaluated. Furthermore, performance changes based on the amount of expansive agent mixed in were compared and analyzed.

## 2. Overview of the Experiment

### 2.1. Experiment

**Figure 1:**
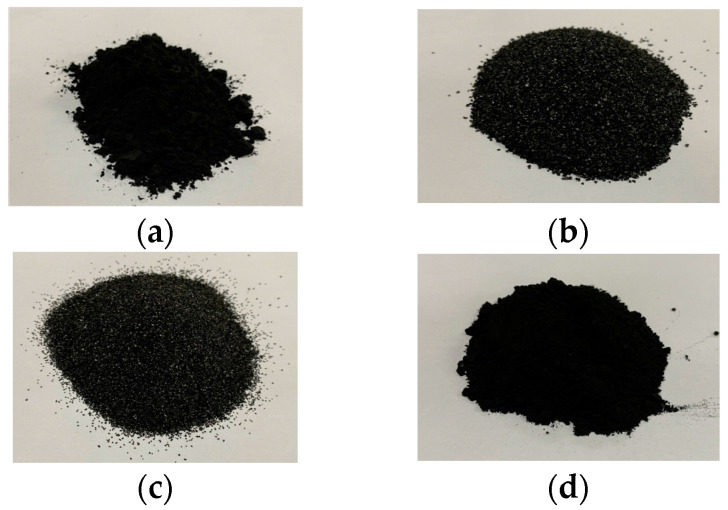
Photographs of carbon materials used as expansive agents in this study: (**a**) oak charcoal, (**b**) palm charcoal, (**c**) broadleaf charcoal, and (**d**) expanded carbon.

In this study, to examine the shrinkage and expansion characteristics of grouts, four types of carbon materials produced in Yangsan, Korea, including oak charcoal (OC), palm charcoal (PC), broadleaf charcoal (BC), and expanded carbon (EC) were used as expansive agents. Figure 1 shows the photographs of the materials used in this study.

To examine the changes in physical performance according to the amount of carbon material that the grout was mixed in, the setting, fluidity, compressive strength, shrinkage, and expansion properties of the mixed material were evaluated, and the results were compared. Carbon materials were used by varying the aggregates by 0.1%, 0.3%, and 0.5%. For comparison, the physical properties of grout mixed with CSA at three concentrations (3%, 4.5%, and 6%) were examined. In addition, to examine the shrinkage and expansion efficiency by material size, the expansion test was performed using OC with seven particle sizes. Moreover, a scanning electron microscope (SEM) was used to examine the pore structure of the carbon materials. The grout mixing procedure followed KS F 4044 (hydraulic cement non-shrink grout). First, cement, fine aggregates, and admixtures were added and mixed for 2 min. Then, water was added and mixed for 1 min. Afterward, the material adhering to the mixing container was scraped off, and the mixture was mixed for an additional 2 min. Table 1 presents the mixing proportions of the grouts.

### 2.2. Materials Used

The cement used in this study was type 1 ordinary Portland cement, specified in KS L 5201, with a specific gravity of 3.15 and a fineness of 3153 cm^2^/g. Table 2 presents the chemical composition of the cement used in this study. The aggregate used was No. 5 silica sand with a specific gravity of 2.61 and an absorption rate of 0.4%. The expansive agents mixed with the grout included CSA and carbon materials (OC, PC, BC, and EC). The chemical composition of CSA is presented in Table 3. Among the carbon materials employed, EC was used in fine powder form without separate sieving. For the other carbon materials, a residue of less than No. 50 sieve was used. Table 4 presents the physical characteristics of the carbon materials used in this study.

### 2.3. Experimental Method

#### 2.3.1. Setting

**Figure 2:**
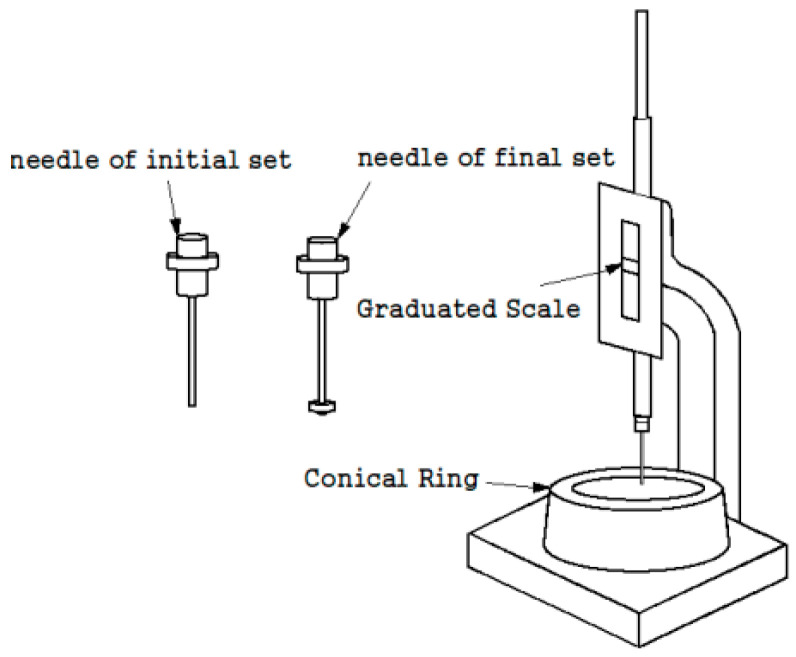
KS L ISO 9597 test method.

The setting time of the grout was measured according to KS L ISO 9597 (The method of testing the setting and soundness of cement) [47], as shown Figure 2. The experimental environment was maintained at a temperature of 20 ± 2 °C and a humidity of 90% or higher. Measurements were performed at 15-min intervals from the beginning of the measurement until the initial setting. Subsequent measurements were performed at 10-min intervals until the final setting.

#### 2.3.2. Fluidity

The fluidity of the grout was measured according to the flow specified in KS F 2476 (test method for polymer cement mortar) [48]. After filling the grout up to the top horizontal surface of the flow cone such that it was flat at the top, the flow cone was lifted vertically. Next, the diameter of the grout spread was measured twice, and the average value was calculated.

#### 2.3.3. Compressive Strength

The compressive strength of the grout was measured according to KS F 2476 (test method for polymer cement mortar) [48]. Three test specimens with dimensions of 40 × 40 × 160 mm were fabricated for each set. The test specimens were cured in water at a temperature of 20 ± 3 °C, and the compressive strength was measured on days 3, 7, and 28 of the curing process.

#### 2.3.4. Shrinkage/Expansion Pre-Hardening

**Figure 3:**
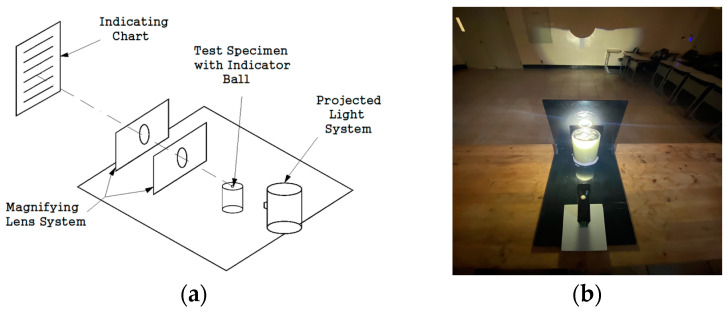
ASTM C 827 test method: (**a**) schematic diagram and (**b**) picture.

To examine the pre-hardening shrinkage/expansion of the grout, it was poured into a Ø 100 × 200-mm acrylic mold. Next, measurements were performed by shining a light from the light source lens and utilizing the enlarged shadow, as shown in Figure 3, in accordance with the specifications of ASTM C 827 (standard test method for change in height at early ages of cylindrical specimens of cementitious mixtures) [49]. The experimental environment was maintained at a temperature of 20 ± 2 °C and a humidity of 60% ± 5%. Measurements were performed at 5-min intervals from the beginning of the measurements until 90 min, at 10-min intervals from 90 min to 150 min, and at 20-min intervals thereafter until the final setting. After the final setting, measurements were performed at 60-min intervals until the end of the 24-h period.

## 3. Experimental Results

### 3.1. Setting Time

**Figure 4:**
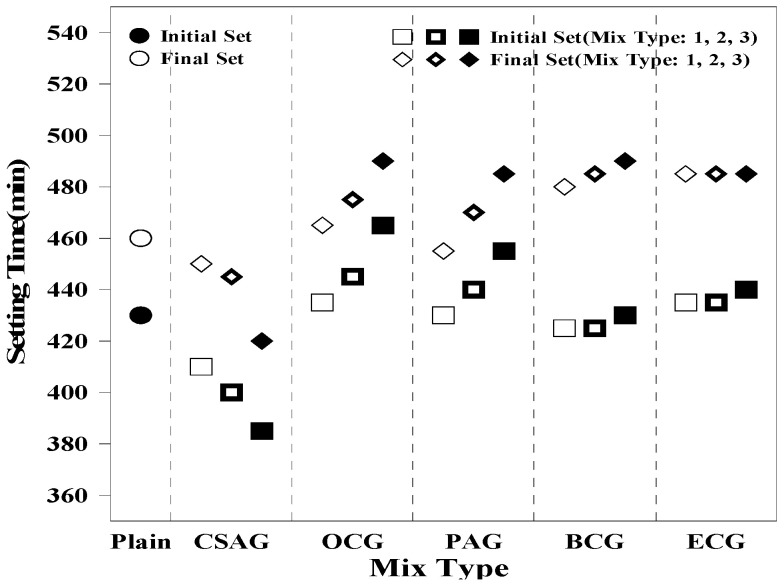
Comparison of the setting times of grouts mixed with carbon materials.

The setting times of grout mixed with carbon materials were compared, and the results are shown in Figure 4. The setting times of all mixing proportions of grout satisfied the criteria specified in KS F 4044 (non-shrink hydraulic grout) [50], which requires an initial setting time of 60 min or more and a final setting time of 600 min or less. In the case of Plain, the initial setting time was 410 min, and the final setting time was 460 min. For CSAG, the initial setting time ranged between 375 and 400 min and the final setting time ranged between 410 and 450 min. These results indicate a tendency for the initial and final setting times to decrease as the mixing ratio of CSA increases. This may be because CSA generated ettringite during the hydration process, thus exhibiting early strength and decreasing the setting time of the grout.

In contrast, for OCG, the initial setting time ranged between 435 and 450 min, and the final setting time ranged between 480 and 485 min. For PCG, the initial setting time ranged between 420 and 430 min, and the final setting time ranged between 470 and 480 min. For BCG, the initial setting time ranged between 420 and 430 min, whereas the final setting time ranged between 475 and 485 min. These results show a tendency for the setting of the grout to be delayed as the mixing ratio of carbon materials is increased. For ECG, the initial setting time ranged between 435 and 440 min, and the final setting time was 485 min. Hence, the effect of the mixing rate was insignificant. Moreover, this study found that none of the grouts mixed with carbon materials were affected by the setting or rapid setting time owing to chemical reactions.

Therefore, it was inferred that the delayed setting of the carbon materials is because of their moisture adsorption characteristics, which induce the gradual hydration of the cement within the grout [51].

### 3.2. Fluidity

**Figure 5:**
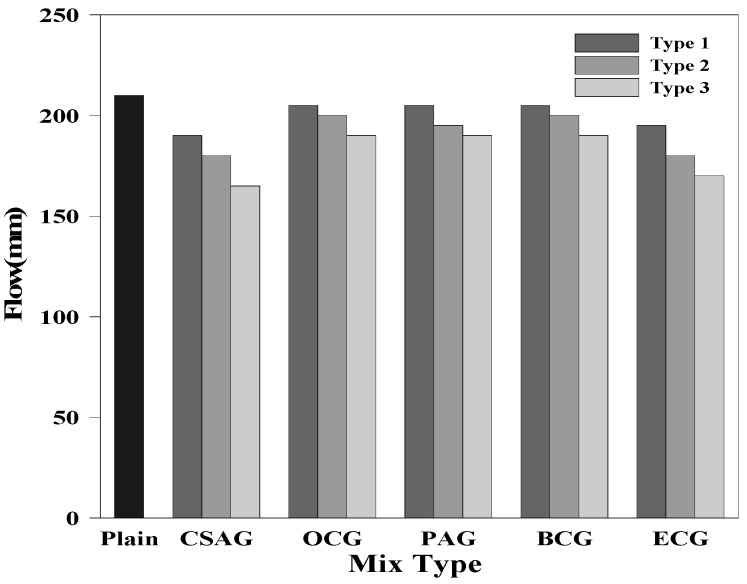
Fluidity of the grouts.

The fluidity test results of the grouts are shown in Figure 5. The flow of Plain was 205 mm, whereas that of CSAG was 190, 180, and 165 mm. Hence, the fluidity decreased as the amount of CSA mixed with grout increased. It was determined that the fluidity of the grout decreased because its viscosity increased due to the high fineness of CSA. In addition, the flow of the grouts mixed with carbon materials is as follows: OCG—205, 200, and 190 mm; PAG—205, 195, and 190 mm; BCG—205, 200, and 190 mm; and ECG—195, 180, and 170 mm. These results showed a tendency for the fluidity to decrease as the amount of carbon materials mixed with grout increased. In the case of the carbon materials with porous structures, it was determined that the moisture absorption rate increased as the mixing ratio increased, which decreased the fluidity [1,51,52,53].

### 3.3. Compressive Strength

**Figure 6:**
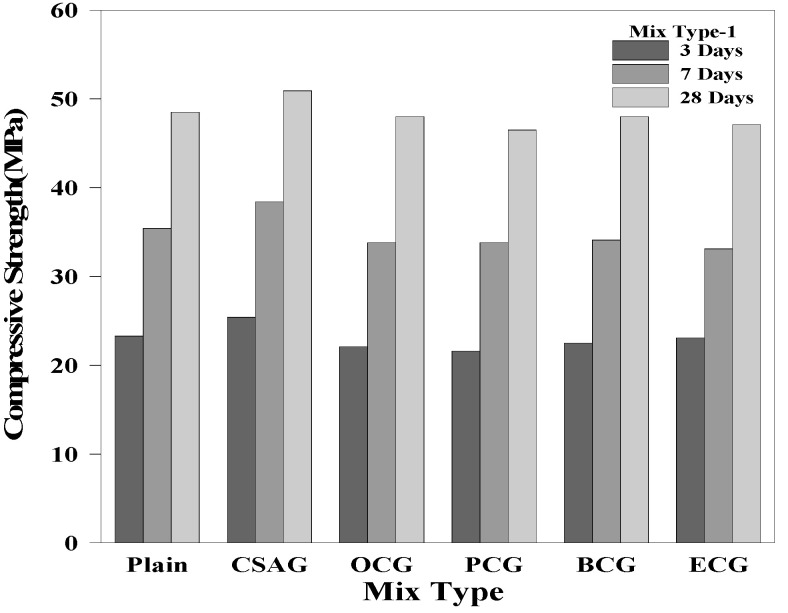
Compressive strength of mix type 1 by age.

**Figure 7:**
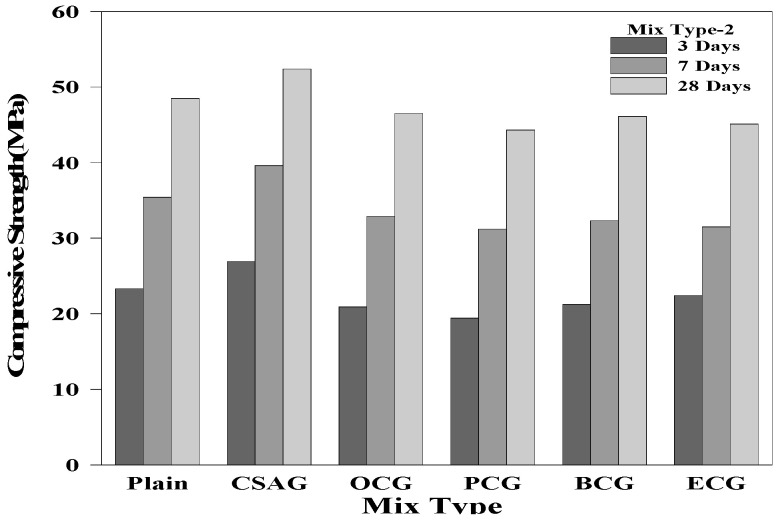
Compressive strength of mix type 2 by age.

**Figure 8:**
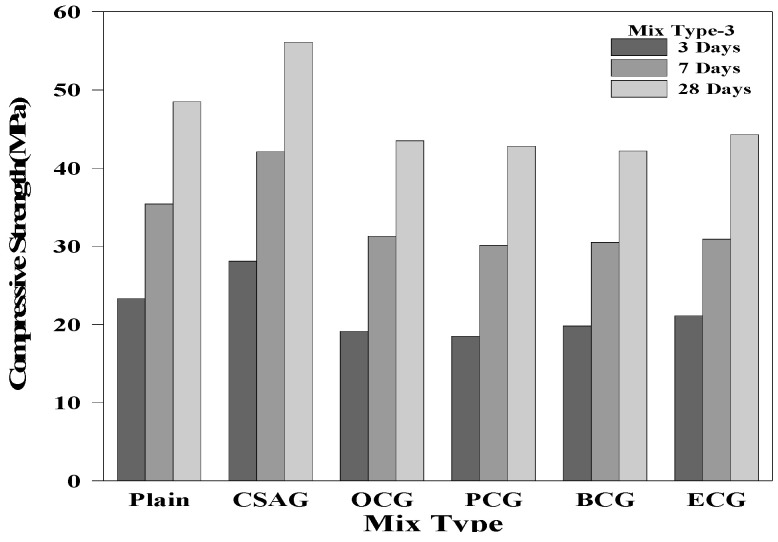
Compressive strength of mix type 3 by age.

Figure 6, Figure 7 and Figure 8 show the compressive strength test results of the grouts mixed with expansive agents. The compressive strength of Plain measured on day 28 of the curing process was 48.5 MPa. For CSAG, the compressive strength on day 28 of the curing process tended to increase from 50.9 MPa and 52.5 MPa to 56.1 MPa, as the mixing ratio of CSA increased. It was believed that the compressive strength increased because the grout became denser as CSA generated a large amount of ettringite [1,3,54,55].

However, the compressive strength of the grouts mixed with carbon materials tended to decrease as the amount of carbon material mixed in increased. Nevertheless, the compressive strength of grouts mixed with carbon materials on day 28 of the curing process exceeded 45.0 MPa, which was close to the compressive strength of Plain. When replacing the cement weight ratio, the strength of the carbon material was lower than that of the cement paste. Therefore, as the replacement rate increases, the strength of the grout decreased. Additionally, the inclusion of carbon materials with porous structures generated air bubbles, creating voids within the grout, which led to a reduction in the compressive strength [12,52,56,57,58,59,60].

### 3.4. Shrinkage/Expansion before Hardening

**Figure 9:**
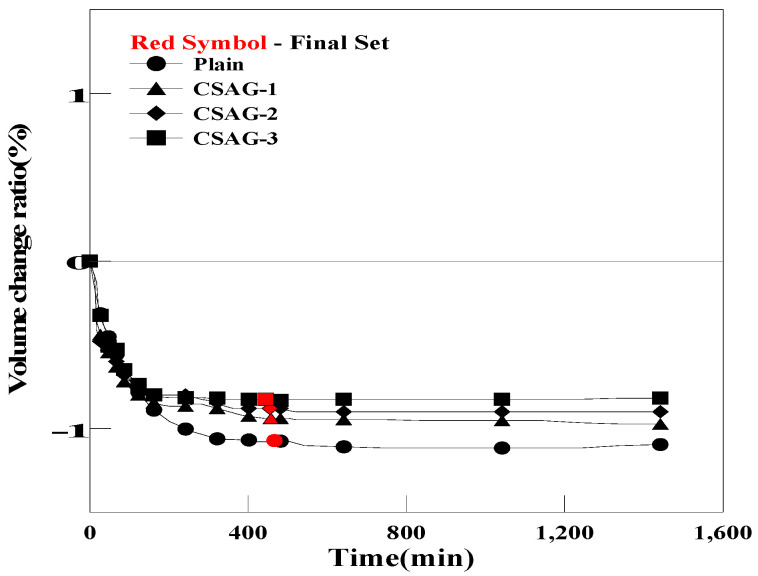
Comparison of the pre-curing expansions of Plain and CSAG.

Figure 9 shows the pre-hardening shrinkage and expansion rates of grout for each mixing ratio of CSA. In the case of Plain, a volume change of −1.07% was observed at the final setting point of grout. For CSAG, volume changes of −0.94%, −0.88%, and −0.82% were observed, depending on the mixing ratio of CSA. As the mixing ratio of CSA increased, the pre-hardening shrinkage of the grout tended to slightly decrease. It was believed that, as the amount of CSA used in the grout increased, the internal moisture was consumed, which reduced the shrinkage of the grout. Moreover, compared with Plain, the difference in the volume changes of CSAG before hardening was insignificant. It was inferred that the ettringite generated by the chemical reaction of CSA did not effectively compensate for the plastic shrinkage that occurred within several minutes to hours during hardening [61].

**Figure 10:**
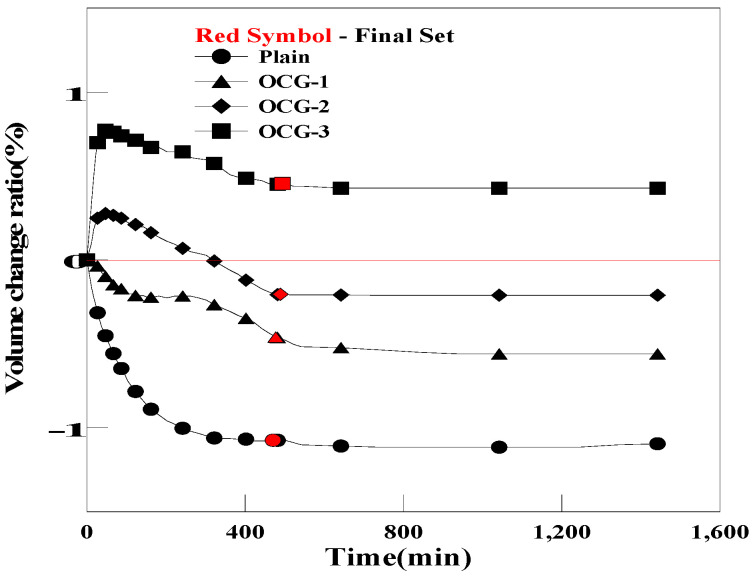
Comparison of the pre-curing expansions of Plain and OCG.

**Figure 11:**
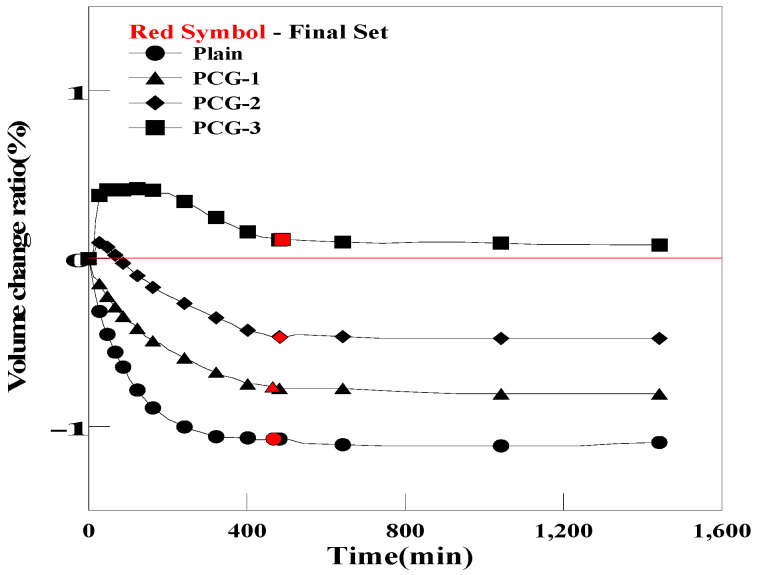
Comparison of the pre-curing expansions of Plain and PCG.

Figure 10 and Figure 11 show the pre-hardening shrinkage/expansion of the grout for each mixing ratio of OC and PC. In the case of OCG, volume changes of −0.43%, −0.20%, and 0.45% were observed, depending on the mixing ratio of OC. For PCG, volume changes of −0.76%, −0.46%, and 0.11% were observed, depending on the mixing ratio of PC. OCG-3 and PCG-3 (a mixing ratio of 0.5% for both OC and PC) expanded more than the initial volume of Plain. This improvement in the expansion performance was achieved because porous carbon materials such as OC and PC absorb moisture when the grout is mixed, and the air contained inside is released, creating bubbles within the grout.

**Figure 12:**
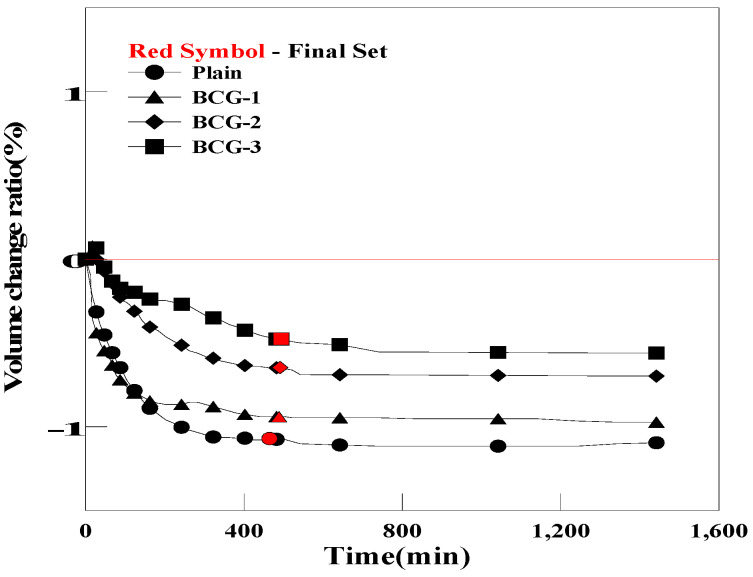
Comparison of the pre-curing expansion of Plain and BCG.

**Figure 13:**
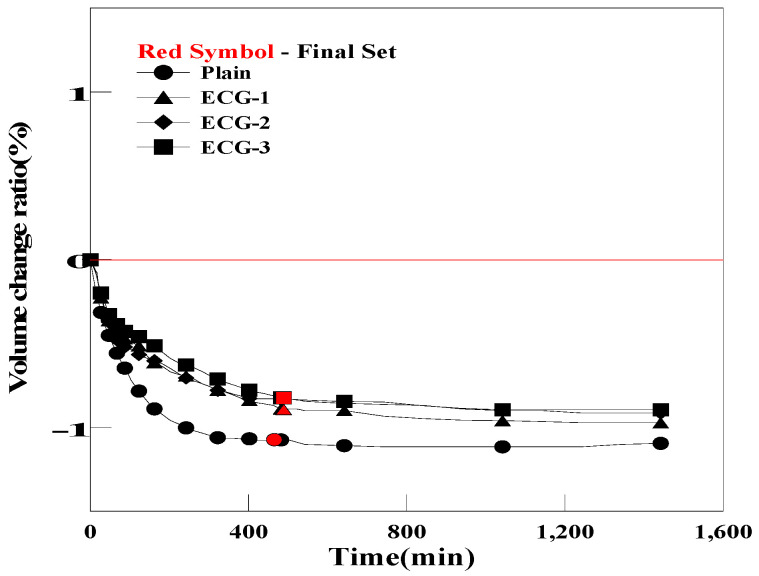
Comparison of the pre-curing expansion of Plain and ECG.

However, as shown in Figure 12 and Figure 13, the grouts mixed with BC and EC exhibited a reduction in the pre-hardening shrinkage. However, volume changes were insignificant compared with those of OCG and PCG [53]. This result can be attributed to the fact that carbon materials with porous structures generate different amounts of bubbles based on the carbon material used, due to differences in the number of micropores and pore size.

### 3.5. SEM Examination of Carbon Materials

**Figure 14:**
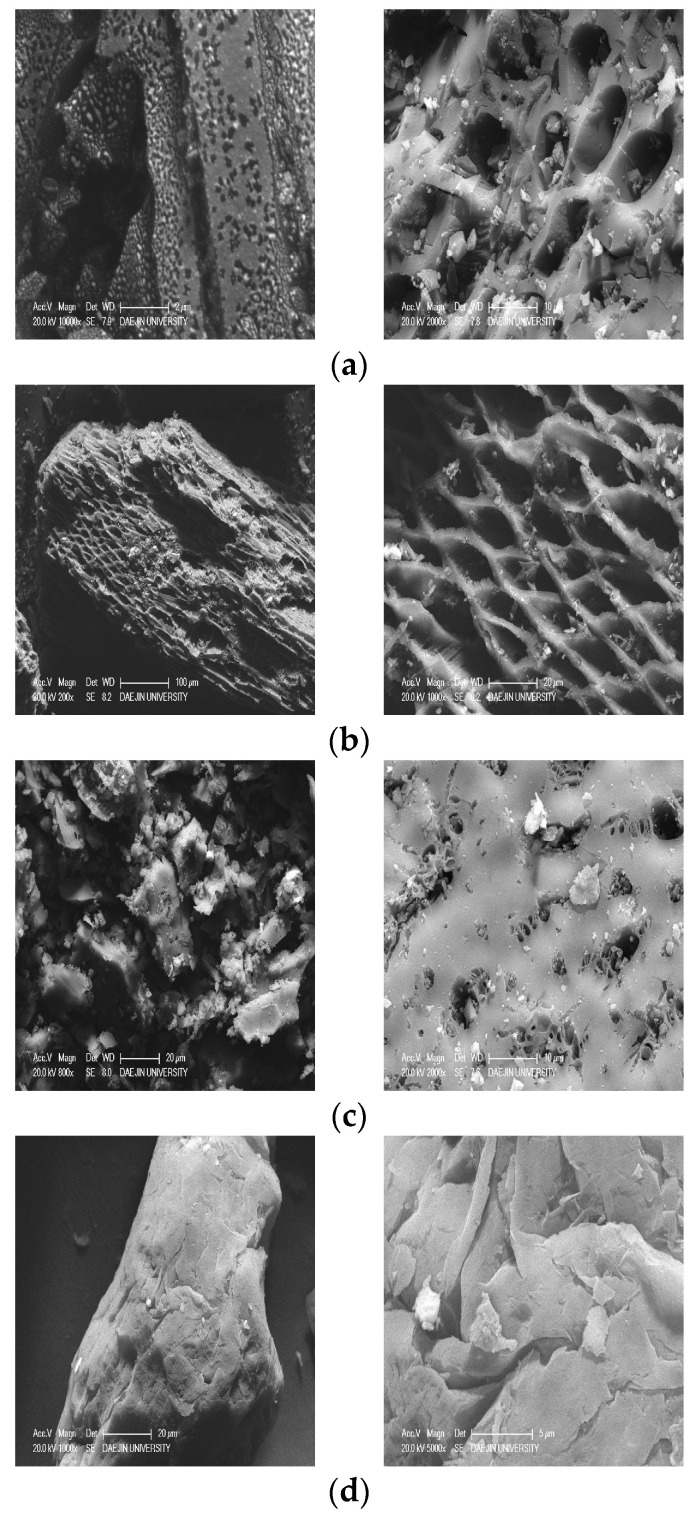
Shape characteristics of the carbon materials: (**a**) oak charcoal, (**b**) palm charcoal, (**c**) broadleaf charcoal, and (**d**) expanded carbon.

Figure 14 shows the photographs of the structures of the carbon materials captured using the SEM. The examination results show that different materials had different pore shapes and sizes. Among the carbon materials, OC and PC had several honeycomb-shaped pores on the surface per unit particle. BC, however, had a relatively small number of pores. The reason for this is that, among the carbon materials, OC, PC, and BC show differences in pore sizes and number of pores due to variations in the carbonization temperature during the charcoal manufacturing process [2]. In the case of EC, pores could not be identified on the surface of the particles. However, EC was identified as a material that expands during the manufacturing process; hence, it was believed that only internal micropores would exist due to the expansion pressure.

### 3.6. Particle Size and Pore Analysis of Carbon Materials

**Figure 15:**
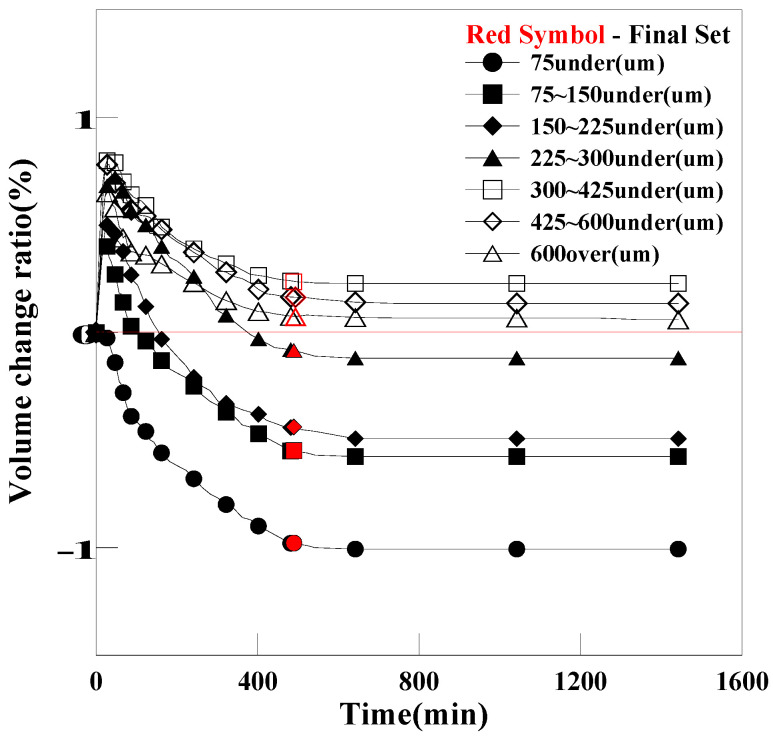
Volume change ratio by particle size of OCG.

To examine the expansion efficiency according to the size of the OC material, seven particle sizes were selected and mixed with the grout. The grout mixed with different particle sizes of OC material is shown in Table 5. The shrinkage/expansion test was conducted on the grouts mixed with the expansive agents of each particle size, and the test results are shown in Figure 15. Particle sizes ranging from less than 75 µm to greater than or equal to 600 µm were examined, and the results showed a tendency for the expansion performance to decrease as the size of the material decreased. The expansion effect was reduced because as the size of the material decreased, the number of pores contained per unit particle was reduced during the grinding process for each material size. The most effective size was found to be between 300 and 425 µm. The expansion test results showed that OC size 2 (≥425 µm) was less effective than OC with particle sizes ranging from 300–425 µm. Hence, it was inferred that there is a limitation on the particle size.

**Figure 16:**
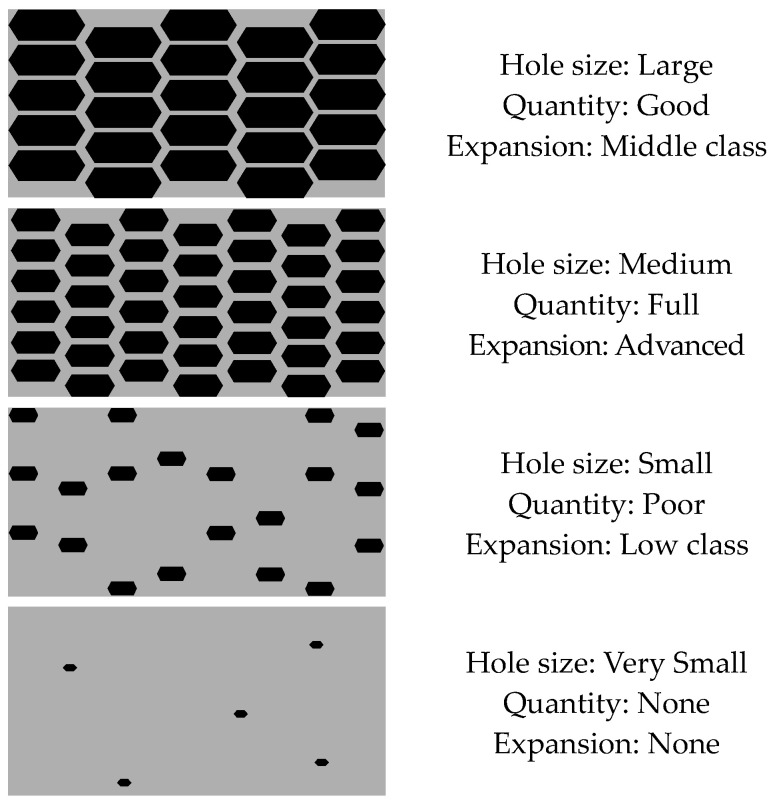
Comparison of expansion performance according to the pore size and quantity.

Therefore, the expansion performance of the carbon materials varied according to the size, number, and length of the pores. The size and number of pores were compared, and four representative shapes were schematically illustrated, as shown in Figure 16 below. 

For the size of the pores, it is important to maintain a certain size. If the pore size is several times larger than the average diameter of 10 µm, both widthwise and lengthwise, air bubbles will be easily released during the mixing process, making it difficult to maintain the expansion performance. However, if the pore size is too small, air bubbles will be released after the cement has hardened, making it difficult to achieve optimal expansion performance.

Finally, the most important and effective condition for expansion performance is having several pores and long lengths of connections inside the pores. However, as shown in the expansion test results for each particle size of the carbon materials, the particle size ranging from 300 to 425 µm was found to be the most efficient for achieving optimal expansion performance. If the internal length of the pores is too long, the efficiency of air emission will be degraded. Therefore, it was determined that the expansion performance would be effective when pores are formed with an appropriate size of 300 to 425 µm.

The structures of BC and EC, which had insignificant expansion performances, had a small number of pores and pores of small sizes. As a result, it was inferred that the expansion performance was insignificant.

## 4. Conclusions

This study investigated the characteristics of cement grouts by mixing them with carbon materials as expansive agents to reduce the pre-hardening shrinkage of cementitious materials. The expansion performance of the grouts mixed with carbon materials was compared with that of the grout mixed with CSA (a conventional expansion agent), and the following conclusions were drawn:

1. The results of the setting test showed that grouts mixed with carbon materials tended to delay the setting time as compared to that of the Plain grout. This outcome can be attributed to the delayed hydration of the cement caused by the moisture adsorption process of porous carbon materials. This outcome is contrary to the outcome of utilizing CSA, which showed a tendency to shorten the setting time.
2. The grouts mixed with carbon materials demonstrated decreased fluidity as compared to the Plain grout. In addition, as the amount of carbon materials that were mixed in increased, the fluidity of the grouts further decreased. The fluidity was reduced because carbon materials with porous structures absorb moisture, which affects the fluidity.
3. The grouts mixed with carbon materials showed a tendency for the compressive strength to decrease as the amount of carbon materials mixed in increased. However, the compressive strength on day 28 of the curing process exceeded 45 MPa, approaching the compressive strength of the Plain grout.
4. In the test results for shrinkage/expansion before hardening, the expansion property of the grout mixed with carbon materials tended to increase as the amount of carbon material mixed in increased. The expansion effect significantly increased in the order of ECG, BCG, PCG, and OCG, according to the materials used. The expansion effect appeared to be closely related to the number and size of pores in the material. Moreover, the expansion effect varied depending on the particle size of the carbon material used. It was found that particle sizes ranging between 300 and 425 µm are most efficient for achieving an optimal expansion performance.
5. The SEM results showed that carbon materials were found to have a porous structure. In addition, OC and PC had several honeycomb-shaped pores on their surfaces. However, BC had relatively fewer pores than OC and PC. In the case of EC, pores on the particle surface could not be observed.

Based on these results, it was determined that using carbon materials as expansive agents could effectively compensate for the pre-hardening shrinkage, which is a drawback of conventional, CSA-based expansive agents. Depending on the application, the amount and size of the pores within the carbon materials can be adjusted to attain the non-shrinking characteristics of hardened cementitious pastes. By securing the non-shrinking characteristics of cement paste, it is expected that this can be applied in various areas such as PSC bridges and machinery foundations. However, further research is needed on the shape, length, and size of the pores within carbon materials, as well as on the expansion performance of materials with a porous structure other than carbon materials. Additionally, performance evaluations will be conducted when incorporating other binders such as ground, granulated blast-furnace slag; fly ash; and silica fume.

## Figures and Tables

**Table 1:** Mixing proportions.

Type	Mix Ratio (Based on Grout)				
	W/G	C	Number 5 SS	Super Plasticizer	Expansion
Plain	19	49.7	50	0.3	-
CSAG-1			47		3
CSAG-2			45.7		4.3
CSAG-3			44		6
OCG-1			49.8		0.1
OCG-2			49.5		0.3
OCG-3			49		0.5
PCG-1			49.8		0.1
PCG-2			49.5		0.3
PCG-3			49		0.5
BCG-1			49.8		0.1
BCG-2			49.5		0.3
BCG-3			49		0.5
ECG-1			49.8		0.1
ECG-2			49.5		0.3
ECG-3			49		0.5

SS: silica sand and W/G: water/grout ratio.

**Table 2:** Chemical composition of ordinary Portland cement.

Material	Chemical Composition (%)						
	SiO_2_	Al_2_O_3_	Fe_2_O_3_	CaO	MgO	SO_3_	LOI
Ordinary Portland cement	15.04	2.48	2.62	73.13	2.00	2.68	2.05

LOI: Loss on ignition.

**Table 3:** Chemical composition of CSA.

Material	Chemical Composition (%)					
	SiO_2_	Al_2_O_3_	Fe_2_O_3_	CaO	MgO	SO_3_
CSA	4.5	12	0.8	51	1.5	30

**Table 4:** Physical properties of the carbon materials used in the study.

Type	Average Particle Size (μm)	Absorption Rate (%)	Specific Gravity
OC	95	16.5	0.725
PC	190	17.4	0.682
BC	155	15.8	0.600
EC	85	13.2	0.086

**Table 5:** Volume change ratio by particle size of OCG.

Material	Volume Change Ratio (%)						
	<75 µm	75–150 µm	150–225 µm	225–300 µm	300–425 µm	425–600 µm	>600 µm
OCG	−0.98	−0.55	−0.44	−0.08	0.24	0.07	0.16

## Data Availability

The original contributions presented in the study are included in the article, further inquiries can be directed to the corresponding author.
